# Peripheral measurements of venous oxygen saturation and lactate as a less invasive alternative for hemodynamic monitoring

**DOI:** 10.1186/s13049-018-0537-7

**Published:** 2018-09-10

**Authors:** Raphaelle Avigael Chemtob, Hasse Møller-Sørensen

**Affiliations:** Department of Cardiothoracic Anaesthesiology, University Hospital of Copenhagen, Rigshospitalet, Blegdamsvej 9, 2100 Copenhagen, Denmark

**Keywords:** Cardiac surgery, Anaesthesia and intensive care, Haemodynamic monitoring, Venous oxygen saturation, Lactate

## Abstract

**Background:**

Peripheral measurement of venous oxygen saturation and lactate is a less invasive alternative to monitor tissue oxygenation as compared to measurements from a central venous catheter. However, there is a lack of evidence to support the use of peripheral measurements. In this study, we investigated the agreement between central and peripheral venous oxygen saturation and lactate.

**Methods:**

We conducted a prospective observational study including 115 patients who underwent elective cardiac surgery between April and May 2015 at Rigshospitalet, Copenhagen, Denmark. Measurements were obtained simultaneously at induction of anaesthesia, upon arrival in the ICU and 3–4 h postoperatively. Bias and trending ability was identified using Bland-Altman analysis and a four-quadrant plot.

**Results:**

Bias was 13.37% for venous oxygen saturation preoperatively (95% CI: 11.52–15.22, LoA: ±19.10, PE: 22.08%), 11.29% at arrival to the ICU (95% CI: 8.81–13.77, LoA: ±25.10, PE: 32.39%) and 16.49% at 3–4 h postoperatively (95% CI: 14.16–18.82, LoA: ±21.20, PE: 26.82%). A four-quadrant plot demonstrated an 89% concordance. Central and peripheral lactate showed a bias of 0.14 mmol/L preoperatively (95% CI: 0.11–0.17, LoA: ±0.30, PE: 32.08%), 0.16 mmol/L at arrival to ICU (95% CI: 0.09–0.23, LoA: ±0.70, PE: 38.88%) and 0.23 mmol/L at 3–4 h postoperatively (95% CI: 0.11–0.35, LoA: ±0.50, 25.18%).

**Discussion:**

Measurements of peripheral oxygen saturation and lactate may be valuable in an emergency setting, avoiding unnecessary and time consuming application of a CVC.

**Conclusion:**

We found a high bias but an acceptable trending ability between central and peripheral venous oxygenation. Central and peripheral lactate had excellent agreement. Further studies are necessary to validate the use of peripheral venous samples to identify patients at risk of impaired tissue oxygenation.

## Background

Adequate tissue oxygenation is necessary to maintain organ function. Venous oxygen saturation reflects tissue oxygenation necessary to maintain normal organ function and is dependent on cardiac output (CO), oxygen concentration of arterial blood (SaO_2_), oxygen carrying capacity (Hb) and metabolic demands of the body (VO_2_) according to Fick’s equation [1].

Low venous oxygen saturation reflects impaired tissue oxygenation and is routinely used as a predictive tool and a therapeutic target to improve the care of critically ill patients [2]. Previous studies have associated impaired venous oxygen saturation with increased mortality and morbidity in both surgical patients [3–9] and patients admitted in the Intensive Care Unit (ICU) [10–20].

Venous oxygen saturation is most commonly measured as central (ScvO_2_) or mixed (SmvO_2_) venous oxygen saturation obtained from a central venous catheter (CVC) or a pulmonary artery catheter, respectively [14]. Measurement of venous oxygen saturation (SpvO_2_) from a peripheral venous cannula is a less invasive alternative to identify patients at risk and help manage patients without a central or a pulmonary artery catheter. However, there is a lack of evidence to support the use of SpvO_2_. A recent study found a moderate agreement between ScvO_2_ and SpvO_2_ measured in patients admitted to the Emergency Department and the ICU. Furthermore, they found central and peripheral lactate levels to have excellent agreement [21]. Only one study has investigated the role of SpvO_2_ as a substitute for ScvO_2_, which has not been assessed in a non-emergent clinical settings before.

We conducted a prospective observational study to compare measurements of venous oxygen saturation and lactate in blood samples obtained from a central and a peripheral venous cannula in patients admitted for elective cardiac surgery. In this study, we hypothesize that peripheral venous sampling can estimate measurements of central venous oxygen saturation and lactate, as a less invasive alternative to measure patient hemodynamic.

## Methods

### Study design

The study was conducted as a prospective single-centre observational study. Patients who underwent elective cardiac surgery, from April 29, 2015 to May 16, 2015 at Copenhagen University Hospital, Rigshospitalet, Copenhagen, Denmark, were included. Patients with a central and a peripheral venous cannula were eligible for the study. Exclusion criteria were age below 18 years and acute surgical patients.

The collection and processing of data were approved by the Danish Data and Protection Agency (j. no: 2012-58-0004). The regional ethics commission of the Copenhagen Region waived the need for informed consent as the study was appreciated as a quality-improvement study.

### Patient management

Surgery was performed through a median sternotomy with patients on cardiopulmonary bypass (CPB). Anaesthesia was induced with propofol 1–2 mg/kg i.v. and fentanyl 5 μg/kg i.v., and maintained with sevoflurane 0.5–3% and remifentanil infusion 0.3–.06 mcg/kg/hour. All patients were intubated and mechanically ventilated with 8 ml/kg, frequency 10–12/min, FiO2 0.6, PEEP 5–10 cmH2O, pCO2 4.5–6.5 kPa. Perioperative treatment of transfusions, fluid or drug infusion was given according to the National Guidelines and routine management at the department.

### Study protocol

The CVC was placed after anaesthesia induction. Routinely a second peripheral cannula was inserted simultaneously with placement of the CVC. The peripheral intravenous site was determined by the treating physician with no input from the study investigators. Venous blood samples were obtained simultaneously from the central and peripheral venous cannula, in the latter after application of a tourniquet and with a maximum of 5 min between the two samples. The peripheral venous cannula site and tourniquet application time was registered. Any fluid or drug infusion was discontinued during blood sampling. Approximately 0.2 mL of blood was collected for analysis of gases after an adequate waste draw of 5–10 mL. Blood samples were obtained at pre-specified time-points: as soon as the CVC was inserted, upon arrival in the ICU and 3–4 h postoperatively. All blood samples were analysed on an ABL 800 FLEX radiometer. Results from the central venous blood gases were available to the treating physician.

### Statistics

A Bland-Altman plot was performed to describe the agreement between the two methods at all three time points. The bias and 95% limits of agreement (LoA) with confidence interval (CI) and the percentage error (PE) calculated according to Bland-Altman for all measurements [22]. The PE was calculated as 1.96 times SD of the bias divided by the mean value of ScvO_2_ and SpvO_2_. The results were categorized according to previous studies defining the agreement between two measurements using Bland-Altman plots [23, 24].

In a sub analysis the preoperative data were divided in to two subgroups (SpvO_2_>/< 93%) to analyse the arterialisation of SpvO_2_ observed in some patients. A four-quadrant plot was constructed to assess the trending ability.

Statistical analyses were performed using SPSS version 22.

## Results

A total of 115 patients were included in the study. The majority of patients underwent coronary artery bypass grafting (50%), valve replacement (26%) and combined coronary artery bypass grafting and valve replacement (11%). The remaining patients underwent other cardiac surgical procedures including myectomy and repair of aortic aneurism (13%). Among the peripheral blood samples, regardless of the time point, 9% were obtained with the use of a tourniquet whereof 4% had a tourniquet time above 5 s. The majority of peripheral samples were obtained from a dorsal vein on the hand (72%) or from a cubital vein (28%). A total of 316 paired measurements of central and venous oxygen saturation and lactate were obtained from three different sampling points (preoperative, upon arrival to the ICU and 3–4 h postoperative).

### Venous oxygen saturation

The mean value of ScvO_2_ and SpvO_2_ was 80% (±7) and 93 (±8) preoperatively, 72% (±7) and 83% (±13) on arrival to the ICU and 71% (±7.5) and 87% (±10) at 3–4 h postoperatively, respectively. Venous oxygen saturation was on average higher when obtained from a dorsal hand vein compared to cubital samples (Table 1).

**Table 1:** Venous oxygen saturation at different sampling locations

	ScvO_2_	SpvO_2_ (cubital vein)	SpvO_2_ (dorsal vein)
Preoperative	79.67 (±6.99)	86.47 (±9.83)	94.56 (±7.13)
Arrival to the ICU	71.77 (±7.02)	78.98 (±11.49)	84.39 (±13.13)
3–4 h postoperatively	70.60 (±7.54)	79.87 (±14.32)	88.19 (±8.39)

**Fig. 1:**
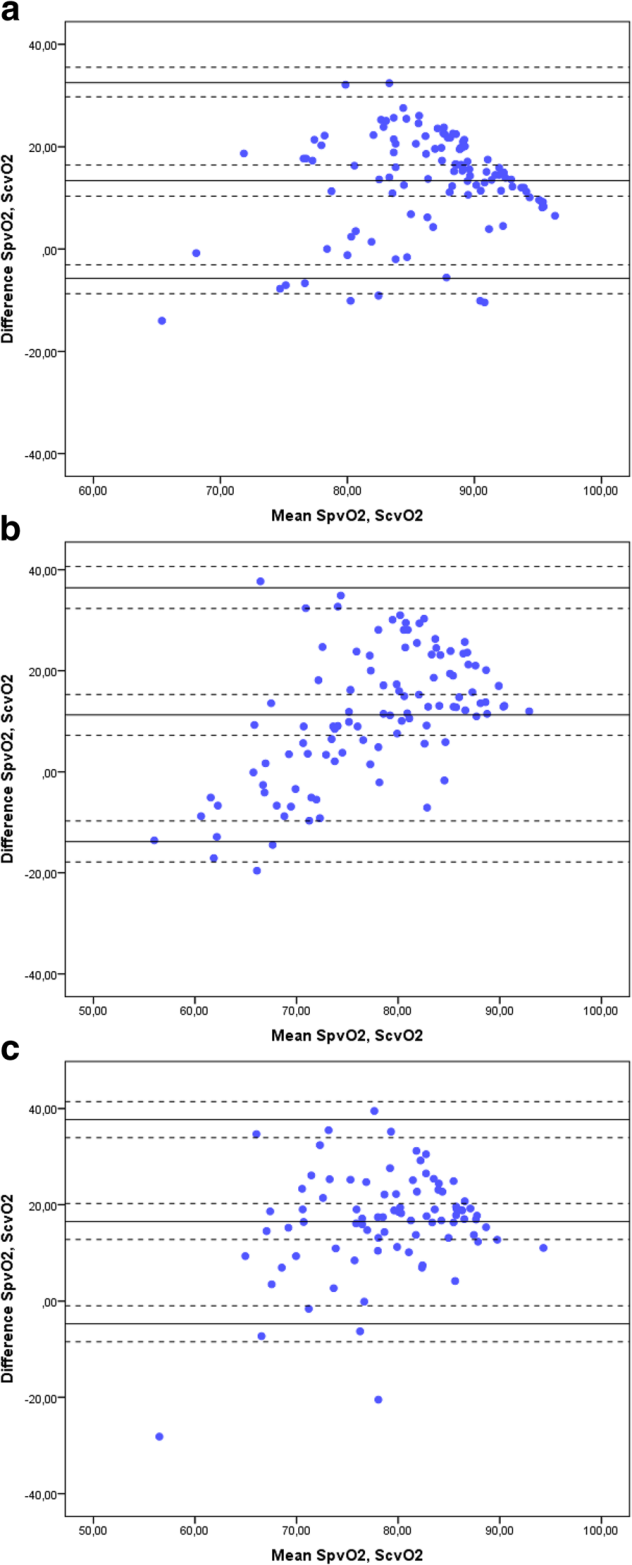
**a** Peripheral and central venous oxygen saturation paired values, differences against means preoperatively. The Bland-Altman plot presents the differences against the means of peripheral and central venous oxygen saturation measurements obtained preoperatively. A total of 114 paired measurements were obtained. Bias was 13.37 (95% CI: 11.52–15.22, LoA: 19.10). The upper and lower LoA are presented with +/− 1.96 SD (95% CI) (dashed lines). The percentage error was 22.08%. Abbreviations: SpvO_2_: peripheral venous oxygen saturation. ScvO_2_: central venous oxygen saturation. **b** Peripheral and central venous oxygen saturation paired values, differences against means at arrival to the ICU. The Bland-Altman plot presents the differences against the means of peripheral and central venous oxygen saturation measurements obtained at arrival to the ICU following surgery. A total of 110 paired measurements were obtained. Bias was 11.29 (95% CI: 8.81–13.77, LoA: 25.10). The upper and lower LoA are presented with +/− 1.96 SD (95% CI) (dashed lines). The percentage error was 32.39%. Abbreviations: SpvO_2_: peripheral venous oxygen saturation. ScvO_2_: central venous oxygen saturation. **c** Peripheral and central venous oxygen saturation paired values, differences against means 3–4 h postoperatively. The Bland-Altman plot presents the differences against the means of peripheral and central venous oxygen saturation measurements obtained 3–4 h postoperatively. A total of 92 paired measurements were obtained. Bias was 16.49 (95% CI: 14.16–18.82, LoA: 21.20). The upper and lower LoA are presented with +/− 1.96 SD (95% CI) (dashed lines). The percentage error was 26.83%. Abbreviations: SpvO_2_: peripheral venous oxygen saturation. ScvO_2_: central venous oxygen saturation

The Bland-Altman plots for venous oxygen saturation are presented in Fig. 1a–c. Bias was 13.37% in the preoperative measurements (95% CI: 11.52–15.22, LoA: ±19.10), 11.29% at arrival to the ICU (95% CI: 8.81–13.77, LoA: ±25.10) and 16.49% at 3–4 h postoperatively (95% CI: 14.16–18.82, LoA: ±21.20). Preoperatively at baseline, 50% of the blood samples had a SpvO_2_ > 93%, indicating arterialisation of blood. For this reason, a sub analysis with the Bland-Altman plot was performed in two separate groups: (SpvO_2_ >/< 93%). Bias was 17.20% (95% CI: 15.80–18.59, LoA: ±11.90) and 4.94% (95% CI: 1.09–8.79, LoA: ±21.60) in the high and the low preoperative group, respectively. The percentage error was 22.08% preoperatively, 32.39% at arrival to the ICU group and 26.83% at 3–4 h postoperatively. The linear regression line was R^2^ =0.12.

**Fig. 2:**
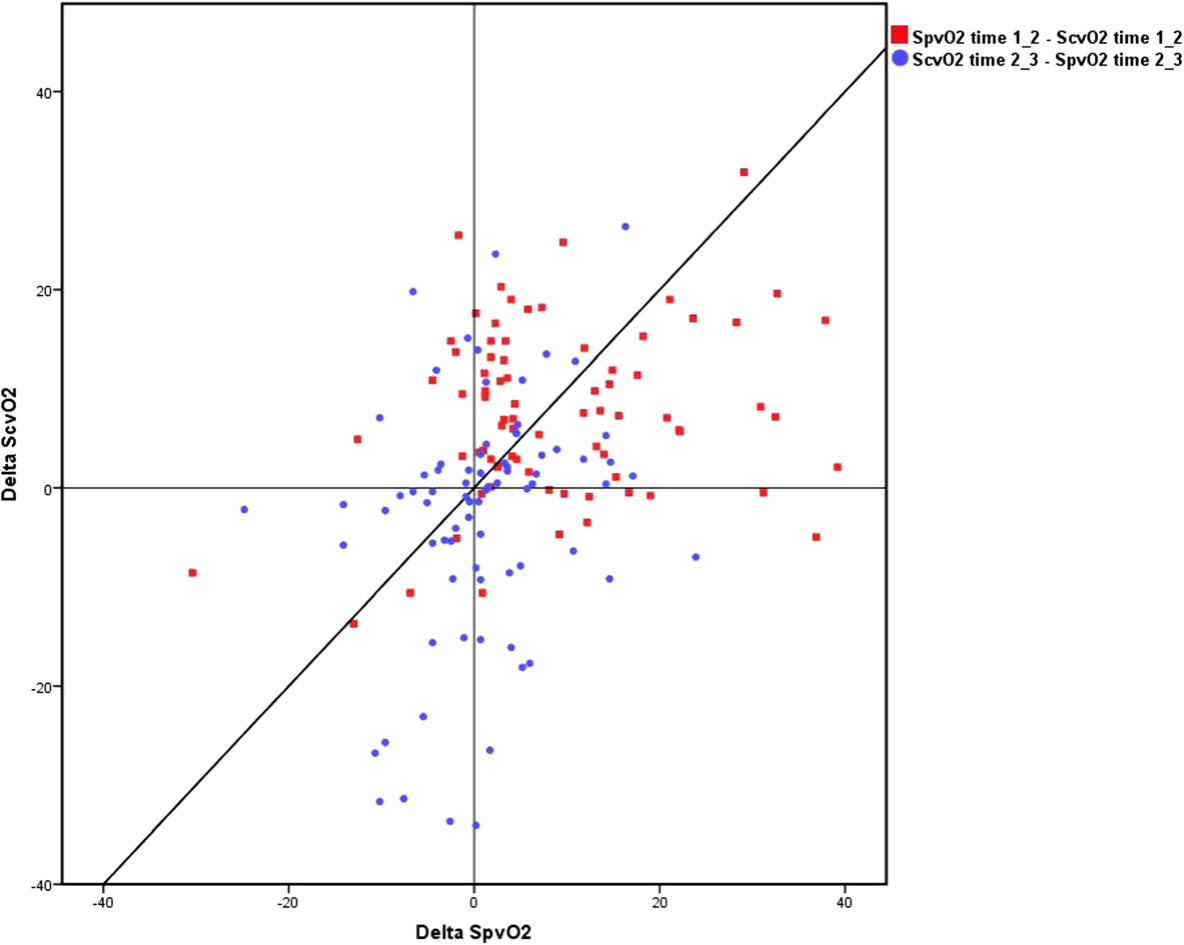
Trending ability of peripheral and central venous oxygen saturation at different time points. Four quadrant plot depicting trending ability of peripheral compared with central venous oxygen saturation between the different sampling points (preoperative, upon arrival to the ICU and 3–4 h postoperative). A total of 316 paired measurements were inserted in the four quadrant plot. The x axis represents the delta SpvO_2_ and the y-axis represents the delta ScvO_2_. Abbreviations: SpvO_2_: peripheral venous oxygen saturation. ScvO_2_: central venous oxygen saturation

The trending ability of ScvO_2_ compared with SpvO_2_ in all measurements was investigated in a four-quadrant plot as demonstrated in Fig. 2. The association between central and peripheral venous oxygen saturation is presented in a scatter plot with a line of identity (Fig. 4a). The plot shows an 89% concordance with no zone of exclusion and 77% concordance including the zone of exclusion.

### Lactate

The mean value of central and peripheral lactate was 0.87 (±0.28) and 1.00 (±0.35) preoperatively, 1.71 (±1.15) and 1.89 (±1.22) on arrival to the ICU and 1.85 (±1.08) and 2.12 (±1.41) at 3–4 h postoperatively, respectively.

**Fig. 3:**
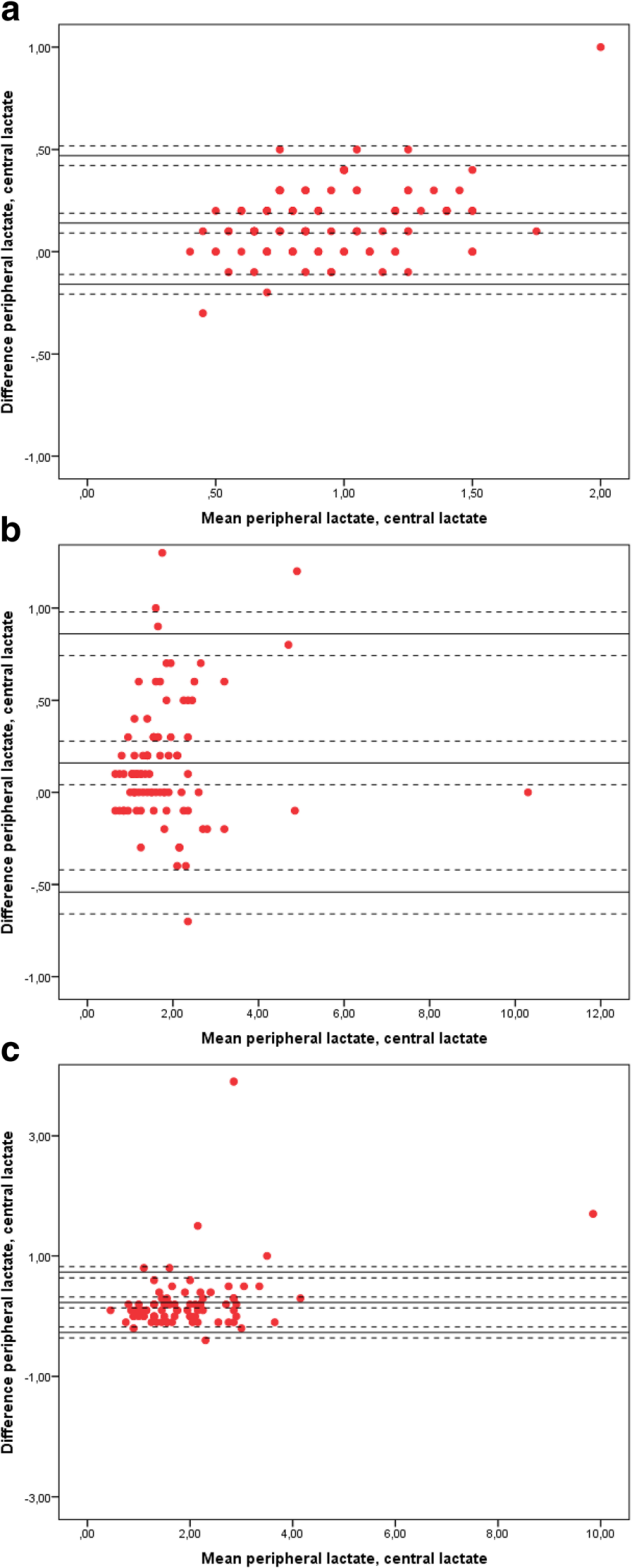
**a** Peripheral and central lactate paired values, differences against means preoperatively. The Bland-Altman plot presents the differences against the means of peripheral and central lactate measurements obtained preoperatively. A total of 111 paired measurements were obtained. Bias was 0.14 (95% CI: 0.11–0.17, LoA: 0.30). The upper and lower LoA are presented with +/− 1.96 SD (95% CI) (dashed lines). The percentage error was 32.08%. **b** Peripheral and central lactate paired values, differences against means upon arrival to the ICU. The Bland-Altman plot presents the differences against the means of peripheral and central lactate measurements obtained upon arrival to the ICU. A total of 100 paired measurements were obtained. Bias was 0.16 (95% CI: 0.09–0.23, LoA: 0.70). The upper and lower LoA are presented with +/− 1.96 SD (95% CI) (dashed lines). The percentage error was 38.88%. **c** Peripheral and central lactate paired values, differences against means 3–4 h postoperatively. The Bland-Altman plot presents the differences against the means of peripheral and central lactate measurements obtained 3–4 h postoperatively. A total of 87 paired measurements were obtained. Bias was 0.23 (95% CI: 0.11–0.35, LoA: 0.50). The upper and lower LoA are presented with +/− 1.96 SD (95% CI) (dashed lines). The percentage error was 25.18%

**Fig. 4:**
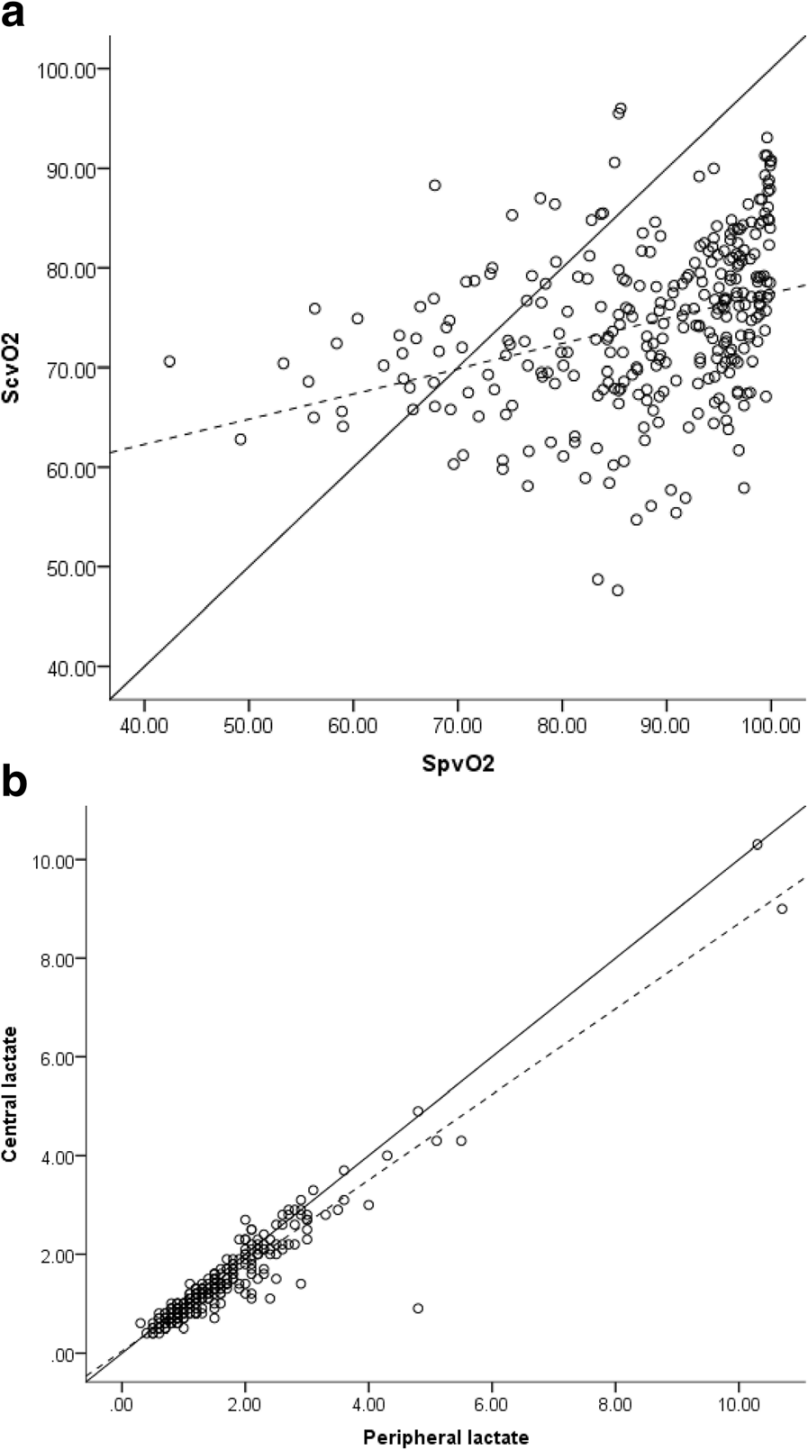
**a** The association between central and peripheral venous oxygen saturation. The scatter plot demonstrates the association between central and peripheral venous oxygen saturation measurements at all three time points. The solid line represents the line of identity and the dashed line represents the linear regression line. *R*^*2*^ = 0.12. **b** The association between central and peripheral lactate. The scatter plot demonstrates the association between central and peripheral lactate measurements at all three time points. The solid line represents the line of identity and the dashed line represents the linear regression line. *R*^*2*^ = 0.89

The Bland-Altman plots for lactate preoperatively are presented in Fig. 3a–c. Bias was 0.14 mmol/L preoperatively (95% CI: 0.11–0.17, LoA: ±0.30), 0.16 mmol/L at arrival to ICU (95% CI: 0.09–0.23, LoA: ±0.70) and 0.23 mmol/L at 3–4 h postoperatively (95% CI: 0.11–0.35, LoA: ±0.50). The percentage error was 32.08% preoperatively, 38.88% at arrival to the ICU group and 25.18% at 3–4 h postoperatively. The association between central and peripheral lactate is presented in a scatter plot with a line of identity (Fig. 4b).

## Discussion

We found a high bias between the measurements of SpvO_2_ and ScvO_2_ at all time-points. However, there was a moderate agreement with a percentage error of 22% to 32%. Furthermore, a moderate trending ability between measurements was found in the present study. A high saturation in peripheral measurements at baseline indicated arterialisation in some patients. Peripheral and central lactate levels showed excellent agreement at all time-points.

A reduction in venous oxygen saturation indicates an increased oxygen consumption/supply-ratio. In the absence of anaemia and arterial hypoxaemia, a low venous oxygen saturation reflects low cardiac output, which may be due to heart failure or obstruction of the circulation as in tamponade or hypovolaemia [3, 25]. Therefore, venous oxygen saturation may be a good indicator of impaired tissue oxygenation [1–3]. Low venous oxygen saturation has previously been associated with impaired outcome in surgical patients, and adequate measures of venous oxygen saturation may have a substantial role in management and treatment of critically ill patients [10].

Placement of a central or a pulmonary artery catheter carries some degree of risk and requires expertise to etablish [26]. Measurements of peripheral oxygen saturation and lactate are beneficial for patients without a central or a pulmonary artery catheter [21]. This would be valuable in an emergency or trauma setting avoiding unnecessary and time consuming application of a CVC in these patients in order to quickly identify patient hemodynamic. We found a high bias indicating a poor correlation between SpvO_2_ and ScvO_2_. However, differences in results between peripheral and central measurements were expected and the objective of the study was not to find a correlation but an association between measurements. Our results showed narrow LoA and a moderate trending ability between measurements, indicating that SpvO_2_ could be a less invasive measure to identify critically ill patients. One study has previously investigated the bias between SpvO_2_ and ScvO_2_ [21] however, the cut-off level for SpvO_2_ has not been estimated. Our results showed higher levels of saturation in SpvO_2_ compared to ScvO_2_. High levels of SpvO_2_ and ScvO_2_ at baseline, indicated arterialisation of the blood. As this was only seen in the measurements obtained at baseline and prior to CPB, this was most probably due to preoxygenation of patients prior to induction of anaesthesia. Arterial dissolved oxygen as a determinant of venous oxygen saturation has previously been described as a potential cause to misinterpretation of ScvO_2_ [27].

Peripheral lactate measurements have previously shown to have agreement with central venous [21] and arterial lactate measurements [28–30]. In accordance with a previous study, peripheral and central lactate showed excellent agreement and peripheral lactate has potential as a substitute for central lactate measures [21]. However, clinical studies are necessary to validate the use of peripheral lactate to identify patients at risk. The association between central and peripheral lactate is presented in a scatter plot with a line of identity (Fig. 3b). The linear regression line was R^2^ =0.89.

### Limitations

Our results showed an acceptable LoA and a moderate trending ability, indicating an acceptable agreement between SpvO_2_ and ScvO_2_. The precision of the individual methods was not determined in this study and the exact value of an acceptable LOA of agreement could not be calculated. However, a percentage error of 22% to 32% is rated to be within the clinically acceptable [23]. Measurements of peripheral venous oxygen saturation and lactate could be valuable in different populations such as emergency or trauma settings, avoiding unnecessary and time consuming application of a central venous or a pulmonary artery catheter, and in order to quickly identify patient at risk and hemodynamics. Routinely all patients get a central and peripheral line prior to cardiac surgery at our department, which allowed us to investigate the feasibility of SpvO_2_ in this patient population. Additional studies are required to investigate whether these observations are reproducible in a different setting. The present study is limited by the fact that only a few patients demonstrated low cardiac output postoperatively and therefore, the clinical implication of SpvO_2_ in patient with low cardiac output is unknown. Surprisingly, a high SpvO_2_ was seen in many patients at baseline measurements. A simultaneously high ScvO_2_ indicated arterialisation in these patients. The risk of arterialisation at different ScvO_2_ values is unknown and should be further investigated. Other limitations to this study were differences in the peripheral intravenous site, which may have altered the results. Tourniquet application in some patients may have caused hemolysis in the peripheral blood samples. However, very few patients had permanence use of a tourniquet during peripheral blood sampling. Venous blood samples were obtained simultaneously from a central or a peripheral venous catheter however, delays may have occurred due to waste drawn.

## Conclusions

To conclude, our result indicated that SpvO_2_ might be a valuable surrogate for ScvO_2_. As for peripheral lactate, measurements showed excellent agreement and is a reliable substitute for central lactate measurements. Additional studies are required to investigate the cut-off level for SpvO_2_ and to clarify whether observations are reproducible in patients with low cardiac output or in emergency settings.

## Abbreviations

CI: Confidence interval
CPB: Cardiopulmonary bypass
CVC: Central venous catheter
ICU: Intensive Care Unit
LoA: Limits of agreement
PE: Percentage error
ScvO_2_: Central venous oxygen saturation
SmvO_2_: Mixed venous oxygen saturation
SpvO_2_: Peripheral venous oxygen saturation
